# Soft tissue restrictors of femoral elevation in direct anterior approach—an anatomic study

**DOI:** 10.1186/s13018-018-1012-x

**Published:** 2018-12-04

**Authors:** Gongyin Zhao, Ruixia Zhu, Shijie Jiang, Chao Xu, Nanwei Xu, Yuji Wang

**Affiliations:** 1 Department of Orthopedics, Changzhou No.2 People’s Hospital, the Affiliated Hospital of Nanjing Medical University, 29 Xinglong Alley, Changzhou City, 213003 Jiangsu Province People’s Republic of China; 2 Nanjing Medical University, 101Longmian Avenue, Jiangning District, Nanjing, 210039 People’s Republic of China; 3 Departments of Orthopedic Surgery and Biochemistry and Molecular Biology, Mayo Clinic, 200 First St. SW, Rochester, MN 55905 USA

**Keywords:** Total hip arthroplasty, Direct anterior approach, Ischiofemoral ligament, Conjoint tendon, Obturator externus, Piriformis, Acetabulum

## Abstract

**Background:**

With the patient in a constant supine position, elevation of the femur in THA (DAA) provides a more intuitive and conducive location of the acetabulum for the correct placement of the acetabular prosthesis, but elevation of the femur for broaching becomes more challenging. The purpose of this study is to analyze the restriction of the ischiofemoral ligament and short external rotation muscles, and its effect on the elevation of the proximal femur in the DAA.

**Methods:**

The study subjects comprised 5 freshly frozen cadavers with 10 normal hips. All of the anatomic dissections of all of the hips were performed through the DAA. The ischiofemoral ligament, piriformis, conjoint tendon, and external obturator were successively resected. All of the proximal femurs of the specimens were levered by a point tip curved retractor that was connected with a dynamometer. Through preliminary measurements, an applied force of 80 N was adopted and maintained on the curved retractor. The experiment was repeated to measure the displacement of the proximal femur being raised after the posterior structures of the hip joint had been resected in a stepwise fashion. The displacement of the retractor was recorded, and the data were then analyzed.

**Results:**

The distance significantly increased after the ischiofemoral ligament was severed (*P* < 0.001). A prominent increase was demonstrated after the conjoint tendons were severed (*P* < 0.001). The distance insignificantly increased after the piriformis was severed (*P* > 0.05). After the obturator externus was cut off, the distance increased by an insignificant amount (*P* > 0.05).

**Conclusion:**

In DAA, the ischiofemoral ligament contributed stability when the femur was being raised. The main contribution of restriction was provided by the conjoint tendon. The tendons of the obturator externus muscle and piriformis muscle did not provide any significant restriction when the femur was being raised.

## Background

The direct anterior approach (DAA) to the hip was initially described in the nineteenth century and has been sporadically used for total hip arthroplasty (THA) [1]. Carl Hueter first described this surgical approach in 1881. Light and Keggi published their extensive experience using this approach for hip arthroplasty in 1980 [2].

As a minimally invasive technique, the DAA for total hip arthroplasty has several advantages compared to previously popular approaches [2]. The hip capsule is surgically accessed without muscle detachment through the muscular interval between the sartorius and rectus femoris medially and the tensor fascia lata (TFL) laterally [3]. The brief supine position was utilized in the DAA so that the accurate orientation of the acetabular cup could be steadily measured. Due to the intermuscular nature and supine position, use of the DAA allows faster patient recovery to ambulation, normal abductor strength, and decreased dislocation rate [4].

Compared with other approaches for THA, the DAA is advantageous in its exposure of the acetabulum and installation of the cup, but because the exposure of the proximal femur is always difficult, the technique has fallen into disuse by many surgeons. Attempts to retract the proximal femur anteriorly have been reported to contribute to proximal femur and femoral shaft fractures [4]. Insufficient exposure always leads to catastrophic consequences, such as prosthesis piercing. The ischiofemoral ligament, piriformis, conjoint tendons, and the obturator externus are all stabilizers of the hip. The ischiofemoral ligament originates from the ischial rim of the acetabulum and inserts itself around the posterior aspect of the femoral neck. Due to its posterior location, it is primarily restricted in its internal rotation [5]. The piriformis originates from the anterior surface of the second to fourth sacral vertebrae and inserts on the crest of the great trochanter. The conjoint tendon connects with the joint capsule medially, connects with the posterior margin of the gluteus medius laterally, and connects with the tendon of the obturator externus inferiorly [6]. The obturator externus, passing like a sling, originates from the external bony margin of the obturator foramen and inserts into the piriformis fossa [6]. As capsular contributions, the conjoint and obturator externus tendons course along the posterior aspect of the hip capsule [7].

As a portion of the short external rotator muscle, the piriformis, conjoint tendon, and obturator externus have different originations and attachments that lead to the different directions of muscle fibers. These different directions of muscle fibers have different functions in the raising of the proximal femur. The aim of this study was to determine the pivotal structures that may be the main restrictors when the hip is being raised in total hip replacement by the DAA.

## Materials and methods

### Subjects

A total of 5 freshly frozen cadaveric specimens (3 men and 2 women) with 10 hips (5 right hips and 5 left hips) were selected in this study. The cadavers had a mean age of 71.8 years (range, 49–89 years). None of the cadavers showed any evidence of previous trauma or surgery to the femur or hip joint. There were no significant differences in the BMI of the cadavers. The approval for this study was obtained from the Institutional Review Board committees of each of the collaborative institutes.

### Measurement of the proximal femur being elevated

**Fig. 1:**
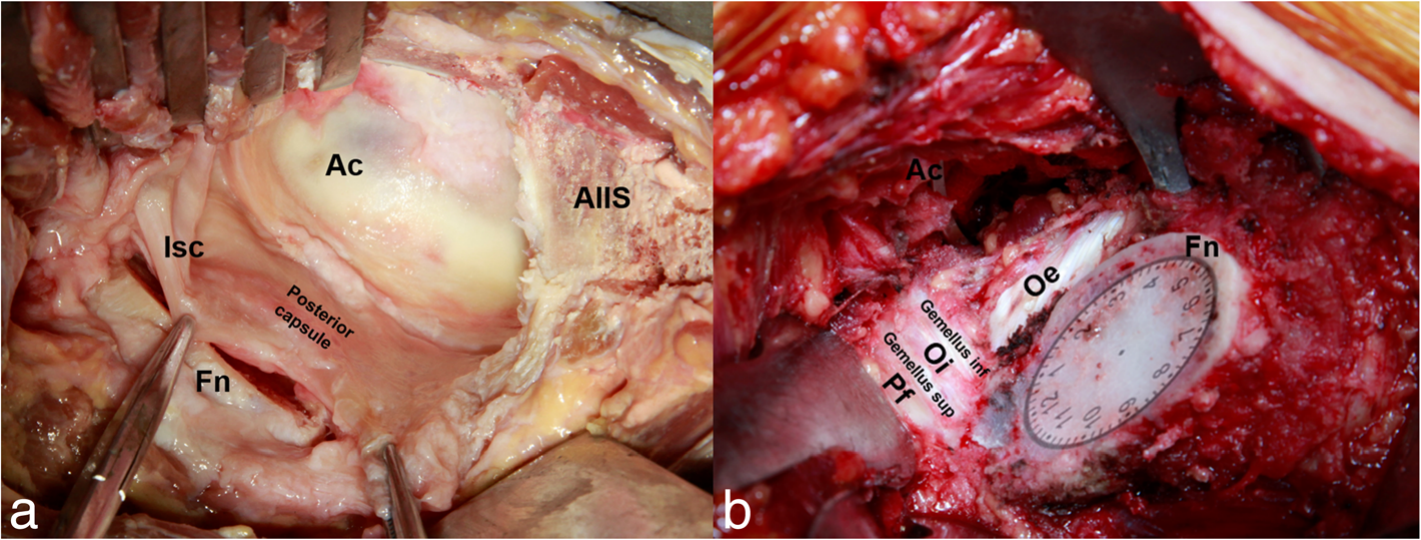
The acetabulum (Ac), femoral neck (Fn), ischiofemoral ligament (Isc), anterior inferior iliac spine (AIIS), and posterior capsule are shown in **a**. The piriformis (Pf), obturator internus (Oi), obturator externus (Oe), and gemellus muscles are shown after ischiofemoral ligament resection in **b**. The clock represents the resected femur, and 12 on the clock indicates the crest of the great trochanter

**Fig. 2:**
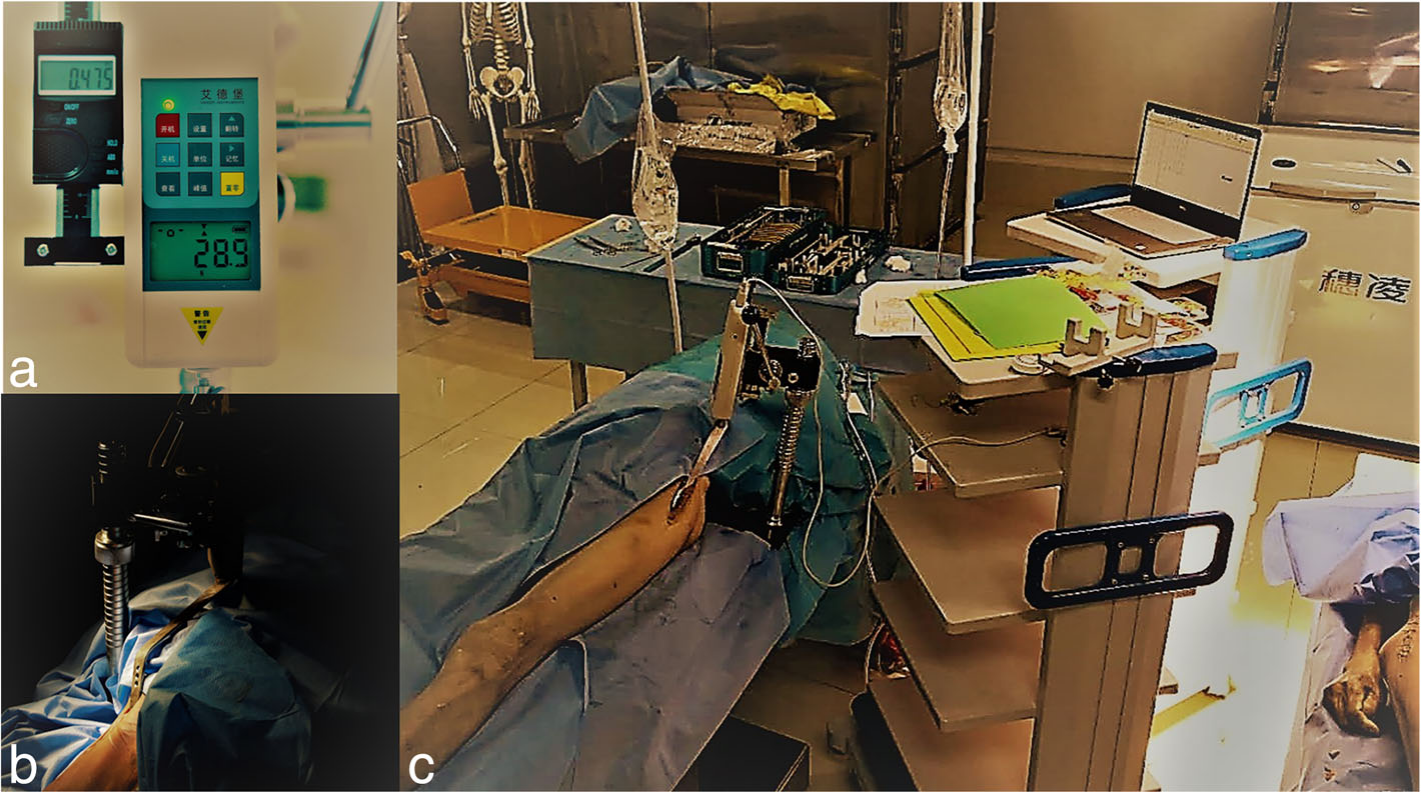
**a** A photo of a dynamometer. **b** The location of the blunt-tipped retractor and the connection with the dynamometer. **c** The entire experimental setup and how the equipment is connected

First, one hip of a cadaveric specimen was randomly selected for anatomic dissection through the DAA. Starting at 2 cm lateral and inferior to the external edge of the anterior superior iliac spine, an incision was made that was 10 cm in length. The space between the TFL and rectus femoris muscle was used as the entrance, and the TFL and rectus femoris muscle were discreetly protected. After the hip joint was distinctly exposed, osteotomy of the femoral neck was implemented. Then, the femoral head was removed by a corkscrew, a blunt tipped retractor was placed at the posterior of the residual femoral neck, and the tail end was installed on a dynamometer. The anterior superior iliac spine served as the fulcrum. For simulating the position of the DAA, the knee was externally rotated at 90°, and the hip was adducted at 20° and extended at 25°. We pressed down the tail of the retractor, then another end of the retractor under the residuary femoral neck was raised up, and the elevation was then recorded [8].We maintained the pressure at 20 N, 40 N, 60 N, 80 N, and 100 N. The corresponding displacement was shown on the dynamometer and recorded when the applied force held steady. Then, the posterior structures of the hip joint were resected in the following order: (1) ischiofemoral ligament, (2) piriformis, (3) conjoint tendon, and (4) obturator externus. We recorded the elevated distance of the femur after these structures were sequentially severed. Figure 1 shows the distinct identification of all of the structures, and Fig. 2 shows structural diagrams.

**Fig. 3:**
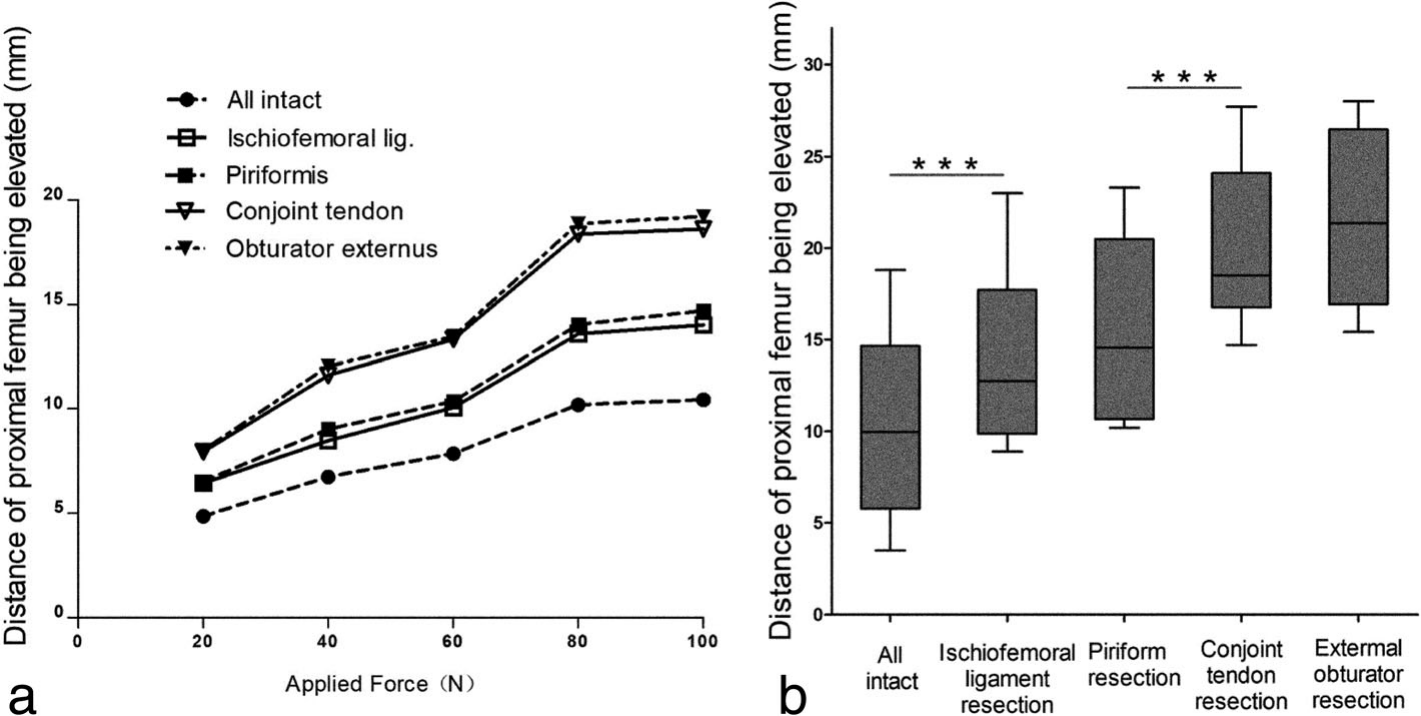
The elevation of the proximal femur simulating the anterior approach with different applied force is shown in **a**. The distance of the proximal femur being elevated simulating the anterior approach at 80 N is shown in **b**. “*” indicates a statistically significant difference

The incremental force caused the increasing elevation of the proximal femur. However, after the pressure exceeded 80 N, the displacement did not significantly increase. When the force was maintained at 80 N, the increasing of the distance was more distinct. The results are demonstrated in Fig. 3a. As a result of the first dissection, it was determined that 80 N was an appropriate pressure and was adopted for the following measurements.

Second, the anatomy of the remaining nine hips was examined by the DAA. The intermuscular interval between the TFL and the rectus femoris muscle was identified, and the removal of the femoral neck proceeded. At 80 N, we repeatedly measured the displacement of the proximal femur being raised after the posterior structures of the hip joint had been resected in the following order: (1) ischiofemoral ligament, (2) piriformis, (3) conjoint tendon, and (4) obturator externus. The data were intensively recorded. All of the piriformis and conjoint tendons were inserted into individual great trochanters so that the tendons could be severed. None of these specimens belonged to the type IV tendon arrangement [9].

### Statistical analysis

The paired *t* test was used to compare the distance of the proximal femur being elevated before and after resection of each structure. *P* values < 0.05 were considered to be statistically significant. The data were analyzed by GraphPad Prism 5 software.

## Results

A total of 10 human freshly frozen cadaveric hips were considered for this study. We applied downward pressure of 80 N after osteotomy of the femoral neck. The distance significantly increased after the ischiofemoral ligament was severed (*P* < 0.001). The piriformis did not provide available restriction when the proximal femur was elevated. (*P* > 0.05). A prominent increase was demonstrated after the conjoint tendons were severed (*P* < 0.001). After the obturator externus was severed, the distance increased by an insignificant amount (*P* > 0.05).

The results demonstrated that the ischiofemoral ligament and a portion of the short external rotator muscle contributed to stability in the hip joint when THA was implemented via DAA. The ischiofemoral ligament and conjoint tendon restricted the proximal femur from being elevated; there was less efficacy with the piriformis and obturator externus when the femur was raised. The results are summarized in Fig. 3b.

**Fig. 4:**
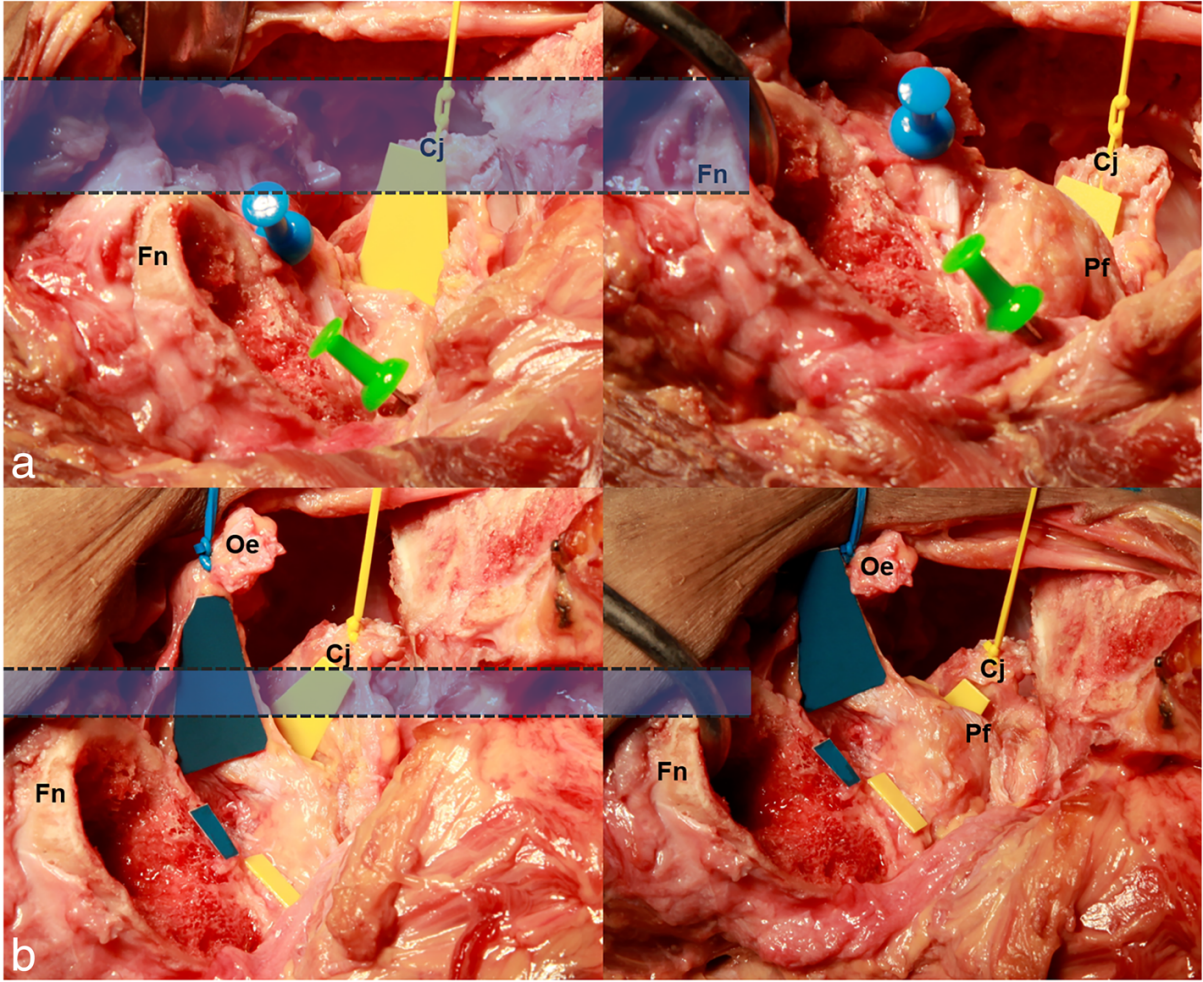
The blue pin represents the obturator externus; the green pin represents the glutes minimus. The yellow trapezoid shape indicates the conjoint tendon; the blue trapezoid shape indicates the obturator externus. The femoral neck (Fn), piriformis (Pf), conjoint tendon (Cj), and the elevation of the femoral neck (blue rectangle) are shown before the obturator externus (Oe) was resected in **a**. The elevation of the femoral neck (blue rectangle) is shown after the obturator externus (Oe) was resected in **b**

The piriformis and obturator externus did not provide restriction when the femur was elevated. A dissection was subsequently carried out, where we reserved the piriformis and then severed the conjoint tendon and the obturator externus in succession. After observing the lateral side, we found that the proximal femur was clearly elevated after the conjoint tendon was severed. However, the elevation rarely increased after the obturator externus was sequentially severed. These observations are demonstrated in Fig. 4, which shows that the height of the blue rectangle represents the distance of the proximal femur being elevated.

## Discussion

When total hip arthroplasty is processed via DAA, the less the proximal femur is elevated, the more difficult the preparation of the femoral canal. Therefore, releasing the posterior structure is important. The ischiofemoral ligament and short external rotation muscle are the main components of the posterior structure. As the main portion of the posterior capsule, the ischiofemoral ligament has been previously described in published studies. It controls the internal rotation in flexion and extension, and because it provides resistance against traction, the ischiofemoral ligament was found to be strong via the anterior and anterolateral approach when the short external rotators of the hip joint are intact [10, 11].

Short external rotator muscles have also been described in many studies. Preservation of the short external rotator muscles is considered to be important because it contributes to joint stability and prevents postoperative dislocation [12]. However, the restriction of the ischiofemoral ligament when the proximal femur is being elevated has not been discussed in previous studies. To date, no experimental study has also focused on the effect of partial or complete restriction of the short external rotator muscles.

In the present anatomical study, we simulated the direct anterior approach by stepwise resection of the ischiofemoral ligament, piriformis, conjoined tendon, and external obturator. The results demonstrated that the ischiofemoral ligament and the conjoint tendon restricted the femur from being elevated. In THA via DAA, to prepare for the release of the femoral portion of the posterior capsule from the greater trochanter, sufficient exposure must be achieved [13–16].

As a strong cylindrically shaped tendon, conjoint tendon consists of the gemellus and internal obturator. The conjoint tendon extends from posteromedial to anterolateral near the greater trochanter, anteriorly to the piriformis tendon and the obturator externus tendon, and is inserted into the medial aspect of the greater trochanter [12]. The conjoint tendon was medially connected with the joint capsule, laterally with the posterior margin of the gluteus medius, and inferiorly with the tendon of the obturator externus [13]. As a sling, the conjoint tendon prevents the greater trochanter from moving toward the outside when the limb is being adducted, and the strong restriction results in an inability of the femur to be elevated.

**Fig. 5:**
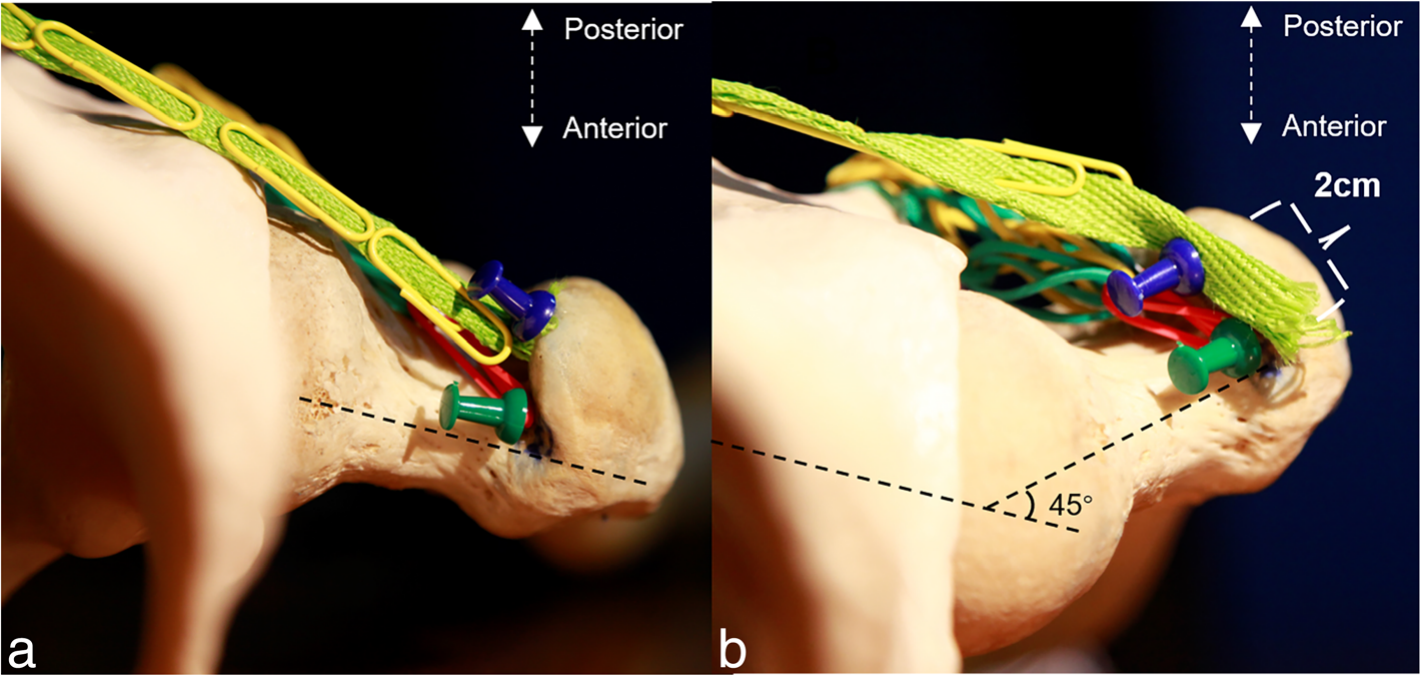
The insertion of the piriformis (blue pin), the insertion of the conjoint tendon (green pin), and the piriformis (green belt) are shown in **a**. After the hip was externally rotated 45°, the 2-cm decrease between the origination to insertion of the piriformis is shown in **b**

As a muscle that did not contribute to the capsule, the piriformis was not important in the process of femur preparation. A model of the hip joint was utilized to demonstrate a simulation of length change of the piriformis. The piriformis originates from the anterior surface of the second to the fourth sacral vertebrae and inserts onto the crest of the great trochanter. In the supine position, the superomedial origination and the inferolateral insertion results in the diagonal muscle fiber being positioned from back to front. When the pelvis is constant and the hip joint is externally rotated, the insertion changes, and the separation from the origination to the insertion is shortened. The shortened distance would compensate for the displacement when the proximal femur was being elevated. As a result, when the lower limb was being externally rotated, the piriformis was flaccid and could not offer a restriction when the femur was being raised. The simulation is demonstrated in Fig. 5. Photo A shows the normal position of the tendons. Photo B demonstrates the situation when the hip joint is externally rotated, and in this photo, the distance was shortened by 2 cm.

The obturator externus tendon extends from medial to lateral, and posteroinferiorly to the obturator internus tendon, and then it inserts into the medial aspect of the greater trochanter, nearly perpendicular to the surface of the greater trochanter [17]. The obturator externus originates from the external bony margin of the obturator foramen and was laterally directed, passing like a sling under the femoral neck to insert into the piriformis fossa through a cylindrical tendon [18]. The origins and the insertions determine the different direction of the tendon fibers. The force provided by the obturator externus acts in a different direction from that of the conjoint tendon. It only limits the movement of internal rotation of the femur and does not provide any restriction when the femur is elevated.

As a minimally invasive surgical method, there is lesser muscle damage, lesser pain, quicker recovery, and better gait mechanics associated with the DAA [19–22]. The anterior superior iliac spine (ASIS), the lateral femoral cutaneous nerve (LFCN), and the TFL as some palpable landmarks were repeatedly mentioned in previous studies [23], but the tendons of short external rotation muscles had not been described in detail. The current anatomical study provides a view of these tendons from the DAA, and the experiments reveal the restriction of the ischiofemoral ligament and conjoint tendon and also indicate that the piriformis and obturator externus did not provide the restriction.

## Conclusions

The ischiofemoral ligament and the conjoint tendon restrict the femoral elevation in DAA and the conjoint tendon plays a major role in this regard. As a well-known muscle, the piriformis is thought to be an important muscle to maintain the stability of the hip joint, but the effect of proximal femur being elevated is limited. The obturator externus is like a cord that restricts the internal rotation of the hip joint only, and it does nothing to limit the elevation of the proximal femur.

Due to the offset broach handle and the fracture table being used, the ischiofemoral ligament and the tendons of the short external rotator muscles could be reserved. However, if the necessary instruments or the fracture table could not be provided, or the proximal femur could not be easily exposed, avoidance of releasing the irrelevant structure and release of the pivotal structure are the important procedures. A better understanding of the arrangement of the short external rotators may improve direct anterior approaches to the hip joint.

This study has a deficiency in that we did not include the influence of the stability of the hip after the conjoint tendon and ischiofemoral ligament had been released, although the dislocation of the hip had not occurred. In the future study, we will try to determine whether the probability of dislocation will be increased after removing the relevant posterior structures.

## Abbreviations

ASIA: Anterior superior iliac spine
DAA: Direct anterior approach
LFCN: Lateral femoral cutaneous nerve
TFL: Tensor fasciae latae
THA: Total hip arthroplasty
